# Diazepam diminishes temozolomide efficacy in the treatment of U87 glioblastoma cell line

**DOI:** 10.1111/cns.13889

**Published:** 2022-06-15

**Authors:** Jovana Drljača, Aleksandra Popović, Dragica Bulajić, Nebojša Stilinović, Sašenka Vidičević Novaković, Slobodan Sekulić, Ivan Milenković, Srđan Ninković, Marko Ljubković, Ivan Čapo

**Affiliations:** ^1^ Department of Pharmacy, Faculty of Medicine University of Novi Sad Novi Sad Serbia; ^2^ Center for Medical and Pharmaceutical Investigations and Quality Control, Faculty of Medicine University of Novi Sad Novi Sad Serbia; ^3^ Department of Physiology, Faculty of Medicine University of Novi Sad Novi Sad Serbia; ^4^ Faculty of Medicine University of Novi Sad Novi Sad Serbia; ^5^ Department of Pharmacology, Toxicology and Clinical Pharmacology, Faculty of Medicine University of Novi Sad Novi Sad Serbia; ^6^ Institute of Medical and Clinical Biochemistry, School of Medicine University of Belgrade Belgrade Serbia; ^7^ Department of Neurology, Faculty of Medicine University of Novi Sad Novi Sad Serbia; ^8^ Department of Neurology Medical University of Vienna Vienna Austria; ^9^ Department of Surgery, Faculty of Medicine University of Novi Sad Novi Sad Serbia; ^10^ Department of Physiology University of Split School of Medicine Split Croatia; ^11^ Department of Histology and Embryology, Faculty of Medicine University of Novi Sad Novi Sad Serbia

**Keywords:** antineoplastic agent, drug interactions, mitochondria, oxidative phosphorylation, U87 glioblastoma

## Abstract

**Aims:**

Many patients with glioblastoma (GBM) suffer from comorbid neurological/psychiatric disorders and, therefore, are treated with psychopharmacological agents. Diazepam (DIA) is widely adopted to treat status epilepticus, alleviate anxiety, and inhibit chemotherapy‐associated delayed emesis in GBM patients. Even though temozolomide (TMZ) and DIA could be found as possible combination therapy in clinical practice, there are no reports of their combined effects in GBM. Hence, it may be of interest to investigate whether DIA enhances the antitumor efficacy of TMZ in GBM cells.

**Methods:**

U87 human GBM was used to examine the effects of combined TMZ and DIA on cell viability, and the oxygen consumption within the cells, in order to evaluate mitochondrial bioenergetic response upon the treatment.

**Results:**

The cooperative index showed the presence of antagonism between TMZ and DIA, which was confirmed on long‐term observation. Moreover, the level of apoptosis after the TMZ treatment was significantly decreased when administered with DIA (*p* < 0.001). Concomitant use of TMZ and DIA increased the basal cell respiration rate, the oxidative phosphorylation rate, and maximal capacity of mitochondrial electron transport chain, as well as the activities of complexes I and II, vs. TMZ alone (*p* < 0.001).

**Conclusion:**

Comparing our results with data reported that DIA elicits cell cycle arrest in the G0/G1 phase and favors senescence reveals that DIA diminishes TMZ efficacy in concomitant use in the treatment of GBM. However, due to its great potency to hinder GBM proliferation and metabolism, it could be considered using DIA as maintenance therapy after TMZ cycles.

## Graphical abstract

Temozolomide exerts its effects through the ability to methylate DNA, which leads to base mismatches and finally resulting in G2/M cell cycle arrest, and ultimately apoptosis. Given that diazepam triggers G0/G1 cell cycle arrest in glioblastoma cells, thus blocking the DNA replication, it diminishes the temozolomide efficacy in concomitant use and should not be administered together.

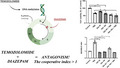

## 1 INTRODUCTION

Gliomas are the most common primary tumors of the central nervous system and represent a heterogeneous group of neoplasms of glial origin. The vast majority of gliomas give rise to glioblastoma (GBM), which is a highly proliferative tumor with invasive growth, pronounced biological heterogeneity, and rated as most severe (gradus IV), according to the World Health Organization.^1^ The global incidence of GBM is <10 per 100,000 individuals and has increased over the last decade. Patients with GBM have a poor prognosis with a 1‐year survival rate of 36.5%, a 5‐year survival rate of 5.1%, and a median survival of ~14 months.^2^

The initial therapeutic approach to GBM is surgical removal, where maximal resection is associated with longer progression‐free survival and overall survival. Patients usually undergo additional radiotherapy and chemotherapy. Concomitant use of temozolomide (TMZ), an oral alkylating agent, significantly increases overall survival in patients with newly diagnosed GBM.^3^, ^4^ However, despite this increase in survival with radiotherapy and TMZ, tumor progression and recurrence are inevitable, due to the development of notorious resistance to TMZ.^5^ Therefore, improved GBM therapy or new therapeutic agents or supplementation of existing therapy is imperative.

Many patients with GBM suffer from comorbid neurological/psychiatric disorders such as headache, motor deficit, anxiety, seizure, dysphasia, and sleep disorders and, therefore, are treated with psychopharmacological agents.^6^, ^7^ It has been reported that 27% of GBM patients present with seizures, due to rapid tumor progression.^8^ Among antiepileptic drugs, benzodiazepines are widely adopted to treat status epilepticus. Besides, benzodiazepines are the first‐choice drug to alleviate anxiety and can inhibit chemotherapy‐associated delayed emesis in GBM patients, as well.^9^, ^10^, ^11^ Having in mind their pleiotropic spectrum of effects, benzodiazepines are frequently prescribed drugs in GBM patients.^6^

Moreover, one of the most commonly used benzodiazepines, diazepam (DIA) has emerged as a promising antitumor agent in various tumors. An increasing number of evidence suggests that benzodiazepine receptors are involved in modulating glioma cells in terms of their energetic metabolism and proliferation and that ligands facilitate apoptosis and cytotoxicity caused by different antineoplastic agents.^12^, ^13^, ^14^, ^15^, ^16^, ^17^, ^18^, ^19^ Several studies reported that DIA inhibited proliferation of rat glioma C6, human melanoma M6, mouse neuroblastoma, breast cancer BT‐20, and rat pituitary tumor cells.^12^, ^20^, ^21^ Besides its antitumor activity, DIA has been shown to facilitate chemotherapy‐induced cytotoxicity.^22^, ^23^

Even though TMZ and DIA could be found as possible combination therapy in clinical practice, there are no reports of their combined effects in GBM. Therefore, it may be of interest to investigate whether DIA enhances the antitumor efficacy of TMZ in GBM cells.

## 2 MATERIALS AND METHODS

Some methods employed in the present study are previously described, confirmed, and published by our group (for references please see Ref. ^24^ and Ref. ^25^), and hence, are briefly outlined here. Detailed description of antibodies (manufacturer and dilution used) and primers (sequences, products’ length, and NCBI accession codes) information are given in Appendix S1.

### 2.1 Cell culture conditions

The U87 cells derived from human glioblastoma (ATCC HTB‐14) were used in this study. The cells were maintained in Modified Eagle Medium, as we described earlier.^25^ All reagents were purchased from Capricorn Scientific GmbH.

### 2.2 Drug treatment

The U87 cells were grown in a complete medium for 24 h prior to the following treatments: vehicle (0.05% DMSO), temozolomide (TMZ), diazepam (DIA), or the combined treatment of TMZ and DIA. The cells were treated for 72 h^26^, ^27^ and then used for different analyses. All drugs were dissolved in sterile DMSO at the final concentration of 100 mM and then stored at −20°C. TMZ was purchased from Sigma‐Aldrich, while DIA was a generous gift by Hemofarm.

### 2.3 Cell viability

The cell viability was determined using the methyl‐thiazolyl tetrazolium (MTT) assay by measuring the activity of mitochondrial succinate‐dehydrogenase, as we described earlier.^24^ The results were presented as percent (%) of viability measured in untreated cells. Two independent experiments were carried out with quadruplicate wells.

### 2.4 Cooperative index

The cooperative index (CI) was calculated according to Riva et al.,^26^ in order to evaluate the effects of combined treatments. Using the % of the metabolic activity reduction obtained from the MTT assay, we compared the sum for each drug to the % obtained from combined treatments, using the following formula: CI = (%DIA + %TMZ)/%(DIA + TMZ). CI values <1 indicate a synergistic effect, CI values =1 indicate an additive effect, while CI values >1 indicate an antagonistic effect.

### 2.5 Long‐term survival assay

For longer‐term observation, a clonogenic assay was performed. Cells were plated into 6‐well plates at a density of 1000 cells per well. Following the different treatment strategies for 72 h, all cells were released free and allowed to be maintained in a fresh medium for another 10 days. The medium was changed every 3 or 4 days. Finally, the cells were fixed with methanol and stained with 0.5% crystal violet solution (Sigma‐Aldrich). Results are presented only qualitatively due to the large number of colonies.

### 2.6 Morphological analyses

Following the treatment, the morphological changes were evaluated by phase microscope observation. Moreover, the cells were stained with classical hematoxylin–eosin staining (H&E). These sections were analyzed using a light microscope (Leica DMLB 100 T) and photographed by a camera (Leica MC 190), as described earlier.^24^ Representative images were captured for each group.

### 2.7 Scratch wound healing assay

Exponentially growing cells were seeded in 6‐well plates at a density of 200,000 cells per well and left for overnight incubation. Following 72 h of treatment, the scratches were made using a 200 μl pipette tip, scraping across the confluent cell monolayer.^28^, ^29^ After rinsing with phosphate buffer saline (PBS), cells were maintained in a serum‐free medium. Cell migration into the wound area was evaluated using light microscopy images taken at 0 h and 24 h. Image analysis was done in ImageJ, using the MRI Wound Healing Tool plug‐in. The percentage of open wound area was calculated by taking open wound area at 0 h for 100%.

### 2.8 Immunofluorescence

To examine the expression of several markers, anti‐vimentin and anti‐Bcl‐2 primary antibodies were used with secondary antibodies linked to Alexa Fluor® 488 (for vimentin) or Alexa Fluor® 555 (for Bcl‐2) (goat, polyclonal, both). Quantification of vimentin expression was expressed as corrected total cell fluorescence (CTCF).^24^ The percentage of anti‐Bcl‐2 positive tumor cells (%[Bcl‐2] + cells) was calculated in five microscopic fields at ×200 magnification. For the details, please refer to the Appendix S1.

### 2.9 Quantitative reverse transcription and PCR (RT‐PCR)

Total RNA was isolated from different treatment groups using trizol (TRIzol Reagent, Invitrogen Ambion) with the following DNAse I treatment (TURBO DNA‐free™ Kit, Invitrogen). Further, 2 μg of RNA was converted to cDNA using the High‐Capacity cDNA Reverse Transcription Kit (Applied Biosystems) according to the manufacturer's instructions. The relative expression of the genes *BAX* and *Bcl‐2* was quantified on Real‐Time PCR 7500 Fast (Applied Biosystems) detection system using the Power SYBR™ Green PCR Master Mix (Applied Biosystems). All reactions were run in triplicates, and relative gene expression was normalized to steady‐state expression of *TBP*, calculations made by using the 2^‐ΔΔCt^ method. For the details, please refer to the Appendix S1.

### 2.10 Analysis of mitochondrial function

To examine the oxygen consumption within the cells after 72 h of different treatment conditions, U87 cells were plated in T75 flasks at a density of 800,000 cells. Following the treatment, cells were collected for measuring basal cell respiration rate, the oxidative phosphorylation (OxPhos) rate, maximal respiration capacity, the activity of complex I, complex II‐fueled respiration, as well as complex IV‐fueled respiration, using the Clark oxygen electrode (Oxygraph, Hansatech Instruments). All these parameters were measured by substrate‐inhibitor titration as we described earlier.^24^ The effective concentration of digitonin for cell membrane permeabilization was 10 μg/ml.

### 2.11 Statistical analyses

All data are expressed as mean *±* standard error of the mean (S.E.M.). The normality of the data sets was assessed by the Kolmogorov–Smirnov test. To determine the existence of significant differences between different treatment conditions, the obtained quantitative data on cell viability, BAX/Bcl‐2 ratio and the expression of Bcl‐2 on immunofluorescence were determined using one‐way analysis of variance (ANOVA), and Tukey test was used for post hoc comparison. Open wound area and the CTCF of vimentin were analyzed with the Kruskal–Wallis test followed by Mann–Whitney *U* test. All tests were done in the SPSS software (v 23.0; IBM), and the *p* value lower than 0.01 was considered significant.

## 3 RESULTS

### 3.1 Effects of combined TMZ and DIA on U87 human glioblastoma viability

**FIGURE 1:**
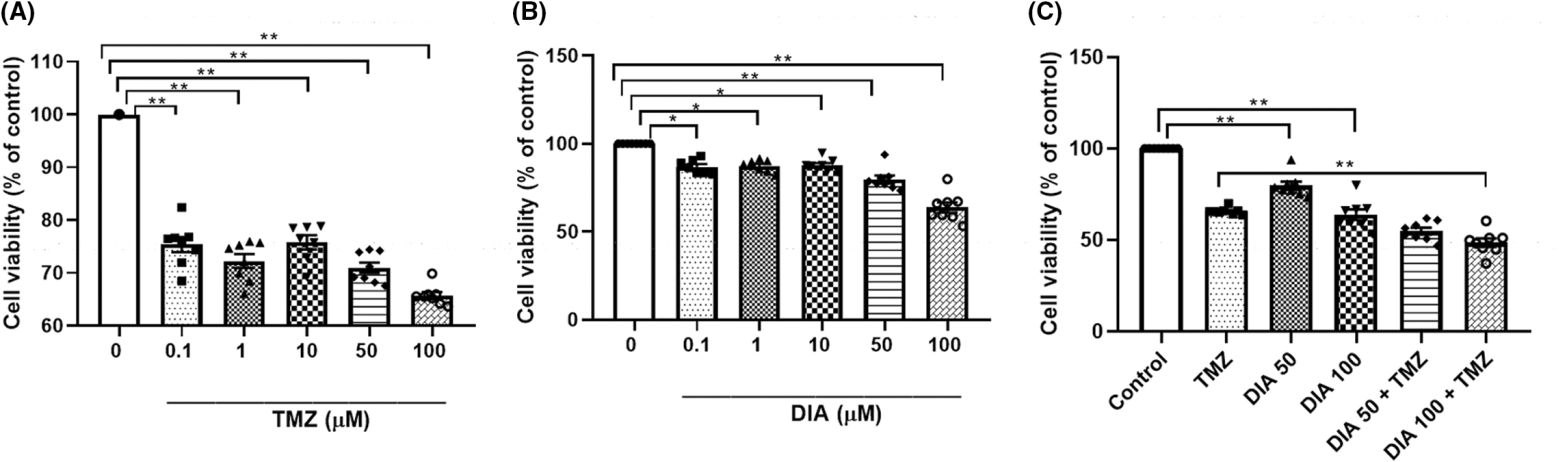
Effects of different concentrations of TMZ (A), DIA (B), or their combination (C) on cell viability in U87 cells. Data are presented as mean ± S.E.M. *p < 0.01 and **p < 0.001.

To establish the effects of TMZ and DIA alone in malignant glioma cells, we treated the U87 human GBM cell line with 0–100 μM TMZ or 0–100 μM DIA for 72 h and assessed the number of viable cells via MTT assay. Both TMZ and DIA showed a growth inhibitory effect in a concentration‐dependent manner (Figure 1A,B). Based on these results, a concentration of TMZ of 100 μM (further referred to as TMZ) and concentrations of DIA of 50 μM and 100 μM (DIA 50, DIA 100), which have been shown as most efficient in the treatment of GBM, were chosen for the following analyses. Therefore, we evaluated the cytotoxicity induced by combined administration of TMZ and DIA after 72 h. As shown in Figure 1C, a significant difference was revealed by one‐way ANOVA (F = 45.504, *p* < 0.001). Further post hoc comparisons showed a significantly enhanced antitumor effect of the combined therapy DIA 100 + TMZ vs. TMZ alone (*p* < 0.001). However, the cooperative index showed the presence of antagonism between TMZ and DIA (CI _DIA 50 + TMZ_ = 1.204; CI _DIA 100 + TMZ_ = 1.363). This finding implies that DIA exposure did not produce any relevant increase in TMZ efficacy in the U87 cell line.

### 3.2 Long‐term survival of U87 cells and the level of apoptosis upon the combined treatment with TMZ and DIA

**FIGURE 2:**
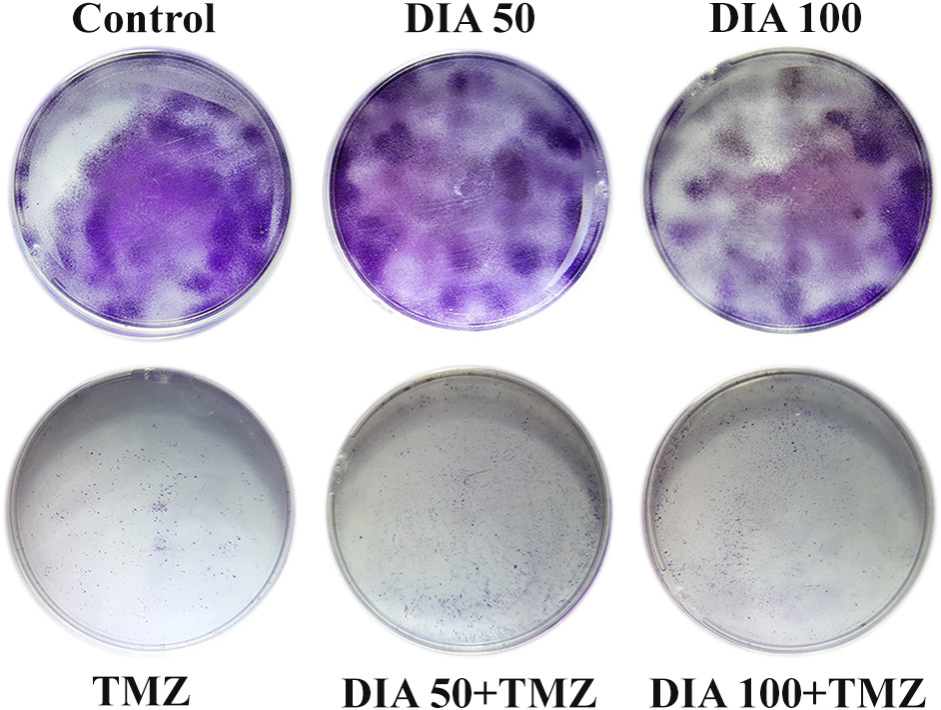
Long‐term effects upon the 72‐h treatment with DIA and TMZ alone or in combination in U87 GBM cells.

For longer‐term observation of the effects of combined therapy, we evaluated the antiproliferative effects 10 days after the treatment (Figure 2). DIA exerted a concentration‐dependent inhibition of glioblastoma cell growth. However, concomitant therapy with TMZ and DIA in both concentrations increased the number of colonies in comparison with TMZ alone group.

**FIGURE 3:**
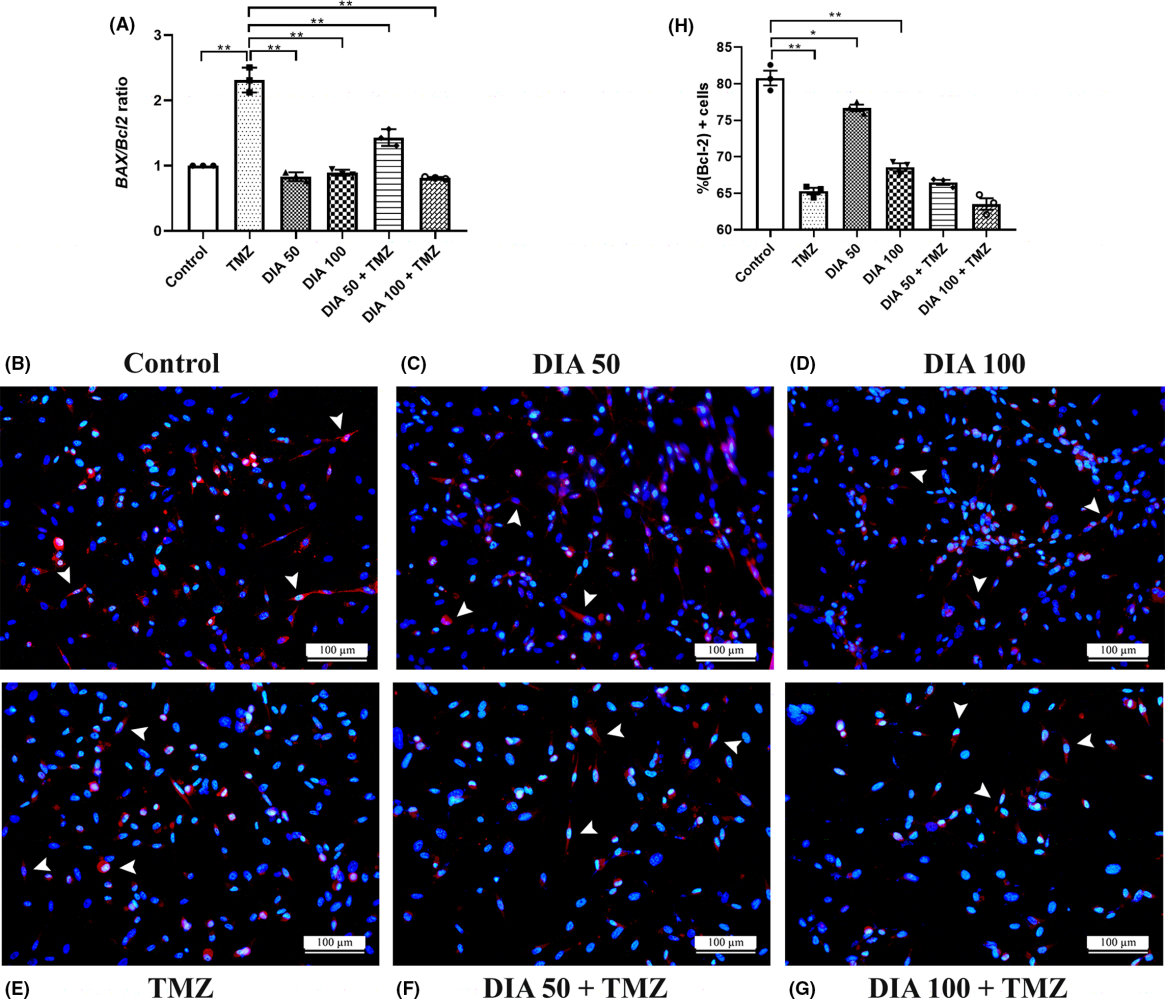
Expression of apoptotic markers in U87 cells. *BAX/Bcl‐2* ratio in cells treated with TMZ and DIA alone, or in a combination (A) immunofluorescence images showing the expression of Bcl‐2 (red) in treated cells (B–G) at a magnification ×200 (arrowheads indicate Bcl‐2 positive cells, and scale bars represent 100 μm), with morphometric analysis of the percentage of Bcl‐2 positive cells upon the treatment (H). Data are presented as mean ± S.E.M. *p < 0.01 and **p < 0.001.

In addition, to gain a better understanding of the combined effects of TMZ and DIA on cell death, we analyzed the ratio of apoptotic markers *BAX* and *Bcl‐2*. The increase in *BAX/Bcl‐2* ratio is thought to contribute to the sensitivity of glioma cells to anticancer therapy by activating the apoptotic cascade.^30^ TMZ alone exerted the highest level of *BAX/Bcl‐2*, which is statistically significant in comparison with other groups (*p* < 0.001), while in concomitant therapy with diazepam its proapoptotic activity was diminished (Figure 3A). Moreover, we performed immunocytochemical analysis to examine the protein expression using the antibody against Bcl‐2 (Figure 3B–G). Further, the percentage of cells expressing antiapoptotic marker Bcl‐2 was determined (Figure 3H), which was significantly decreased following the treatment with DIA 50 and DIA 100 in comparison with untreated cells (*p* = 0.007 and *p* < 0.001, respectively), suggesting a proapoptotic effect of DIA in glioblastoma cells. However, the effects of TMZ and DIA on simultaneous administration did not show a significant decrease in comparison with TMZ alone.

### 3.3 Morphological alterations of U87 cells upon the combined treatment with TMZ and DIA

**FIGURE 4:**
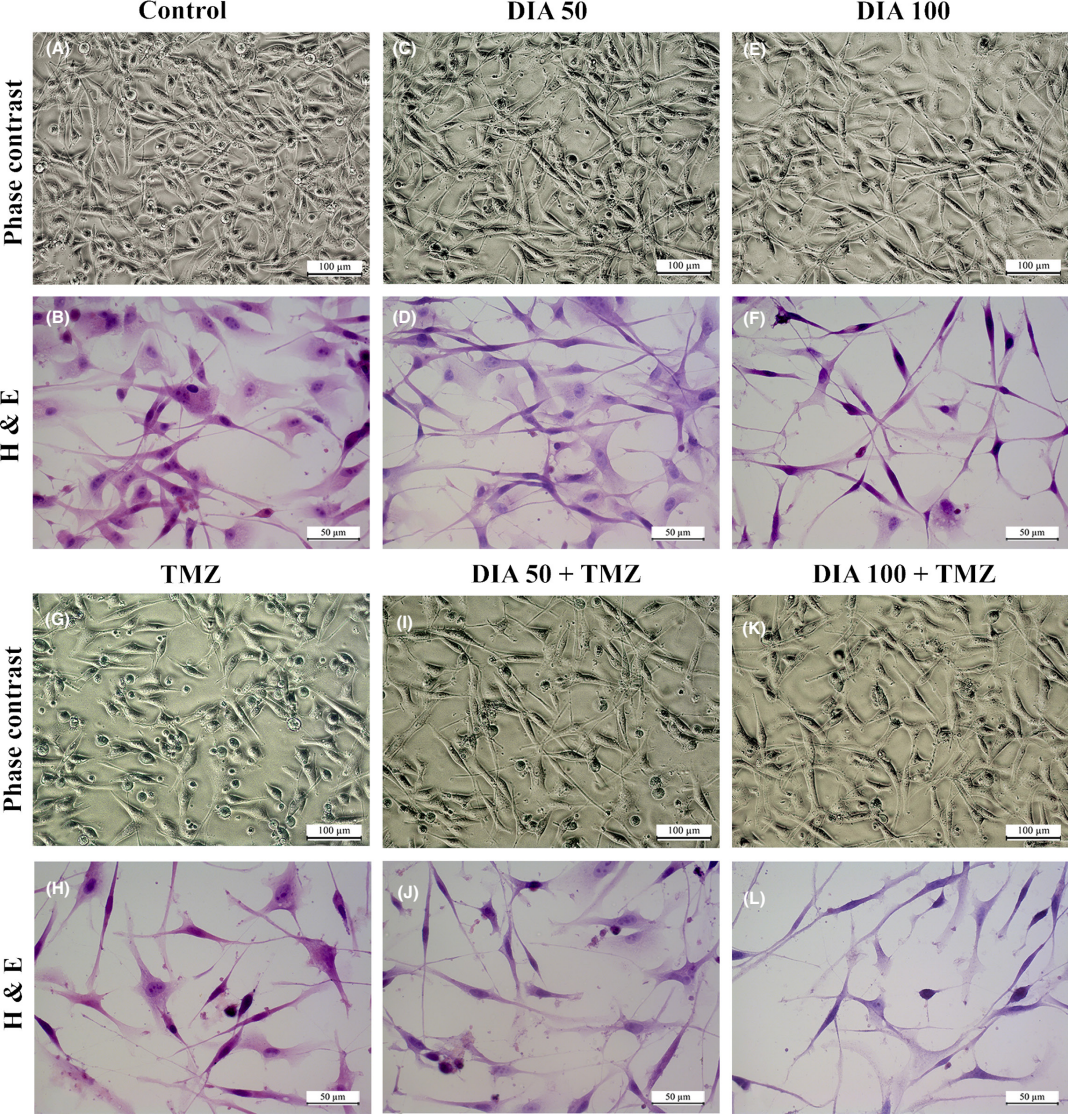
Morphological alterations of U87 cells upon the treatment. Phase contrast (×200 magnification) and H&E staining (×400 magnification) of morphological features of untreated cells (A, B) and upon the treatment with 50 μΜ diazepam (C, D) 100 μΜ diazepam (E, F) and 100 μΜ temozolomide (G, H) alone or in combination (I–L). Scale bars represent 100 μm for phase‐contrast images and 50 μm for H&E stained images.

The morphological alterations of U87 cells were examined after 72 h of treatment using a phase‐contrast microscope and classical H&E staining. Untreated U87 cells possessed heterochromatic nuclei with 3–5 large and centrally positioned nucleoli, showing high pleiomorphism, appearing epitheloid or fusiform, with small branches (Figure 4A,B). After the treatment with DIA, the U87 cells were shrunken with longer protrusions, resembling a neuronal morphology (Figure 4C–F). TMZ‐treated cells became rounded up with predominantly raised nuclear region with condensed chromatin, or elongated with long protrusions (Figure 4G,H). The combined effects on cellular morphology such as cell shrinkage or rounding with dense cytosol were observed in cells treated with the combination of TMZ and DIA (Figure 4I–L).

### 3.4 Migratory capability of U87 cells upon the combined treatment with TMZ and DIA

**FIGURE 5:**
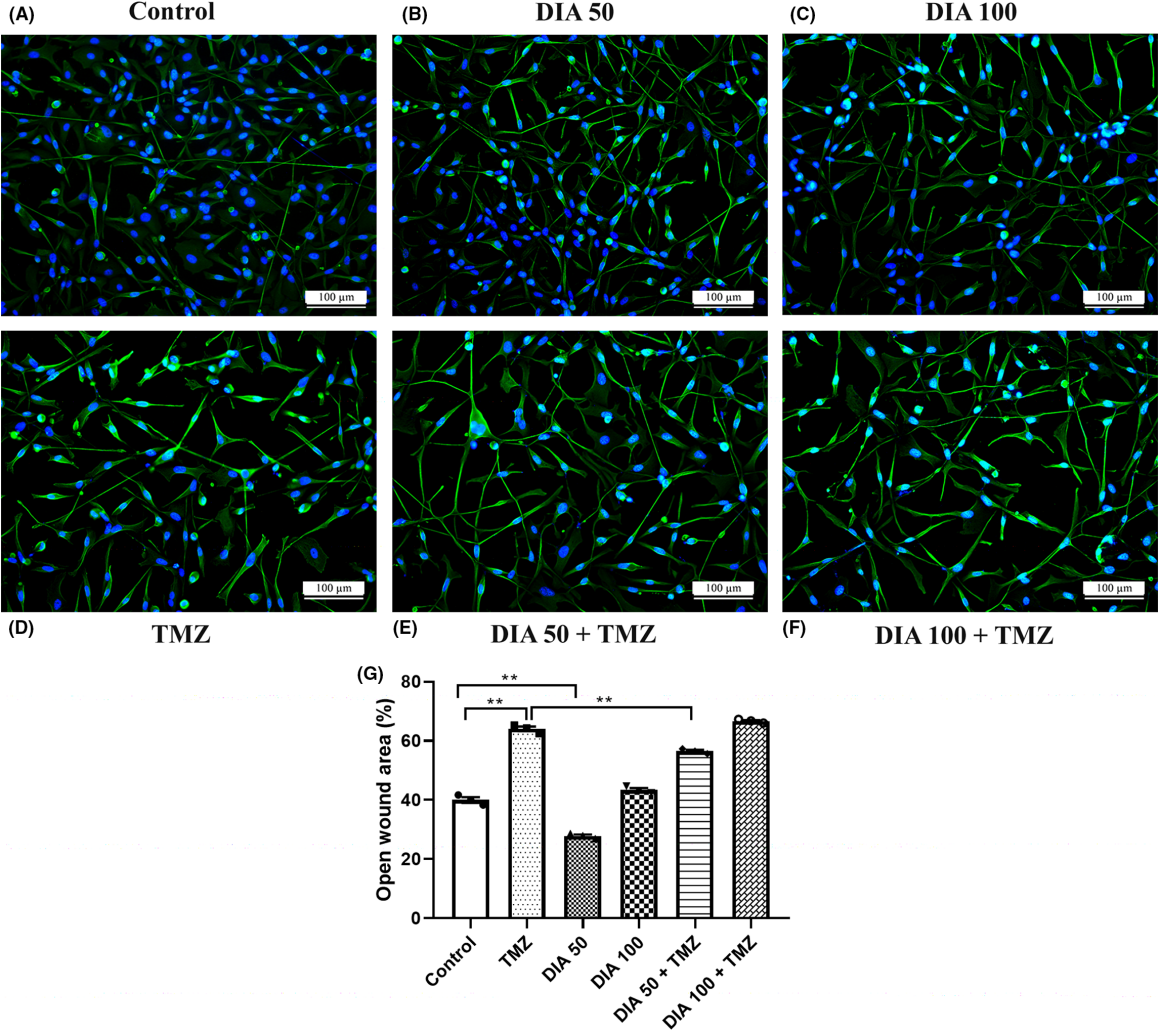
Expression of intermediate filament vimentin and migratory capacity of U87 cells. Immunofluorescence images (at a magnification ×200) showing the expression of vimentin (green) in untreated cells (A), cells treated with 50 μΜ (B) and 100 μM diazepam (C), 100 μM temozolomide alone (D), and in combination with 50 μΜ (E) and 100 μM diazepam (F), with the open wound area % following the treatment (G). Scale bars represent 100 μm. Data are presented as mean ± S.E.M. **p < 0.001.

In order to evaluate whether TMZ and DIA in combination modify the migration capabilities of U87 cells, we performed immunocytochemical staining with the anti‐vimentin antibody and determined the CTCF of vimentin. These results corroborated the morphological alterations in treated cells, displaying changes in the arrangement and appearance of intermediate filament in comparison with control (Figure 5A–F). Despite the increase in CTCF of vimentin in all treated groups, statistical analysis did not reveal any significant differences between the groups (data not shown).

In addition, a scratch wound healing assay was performed (Figure 5G). In order to avoid an increase in the number of migrating cells due to their proliferation, cells were maintained during the experiment in a serum‐free medium. As expected, the migration capacity of the GBM cells was significantly decreased following 72 h of incubation with TMZ (*p* < 0.001), while DIA 100 group did not show a significant difference in comparison with the control, nor did the group DIA 100 + TMZ in comparison with TMZ‐treated group. However, DIA in a concentration of 50 μM significantly increased cell migration in comparison with the rest of the groups (*p* < 0.001). Moreover, the open wound area decreased significantly in the group DIA 50 + TMZ in comparison with the TMZ‐treated group (*p* < 0.001).

### 3.5 Alterations in mitochondrial bioenergetic response upon the combined treatment with TMZ and DIA

**FIGURE 6:**
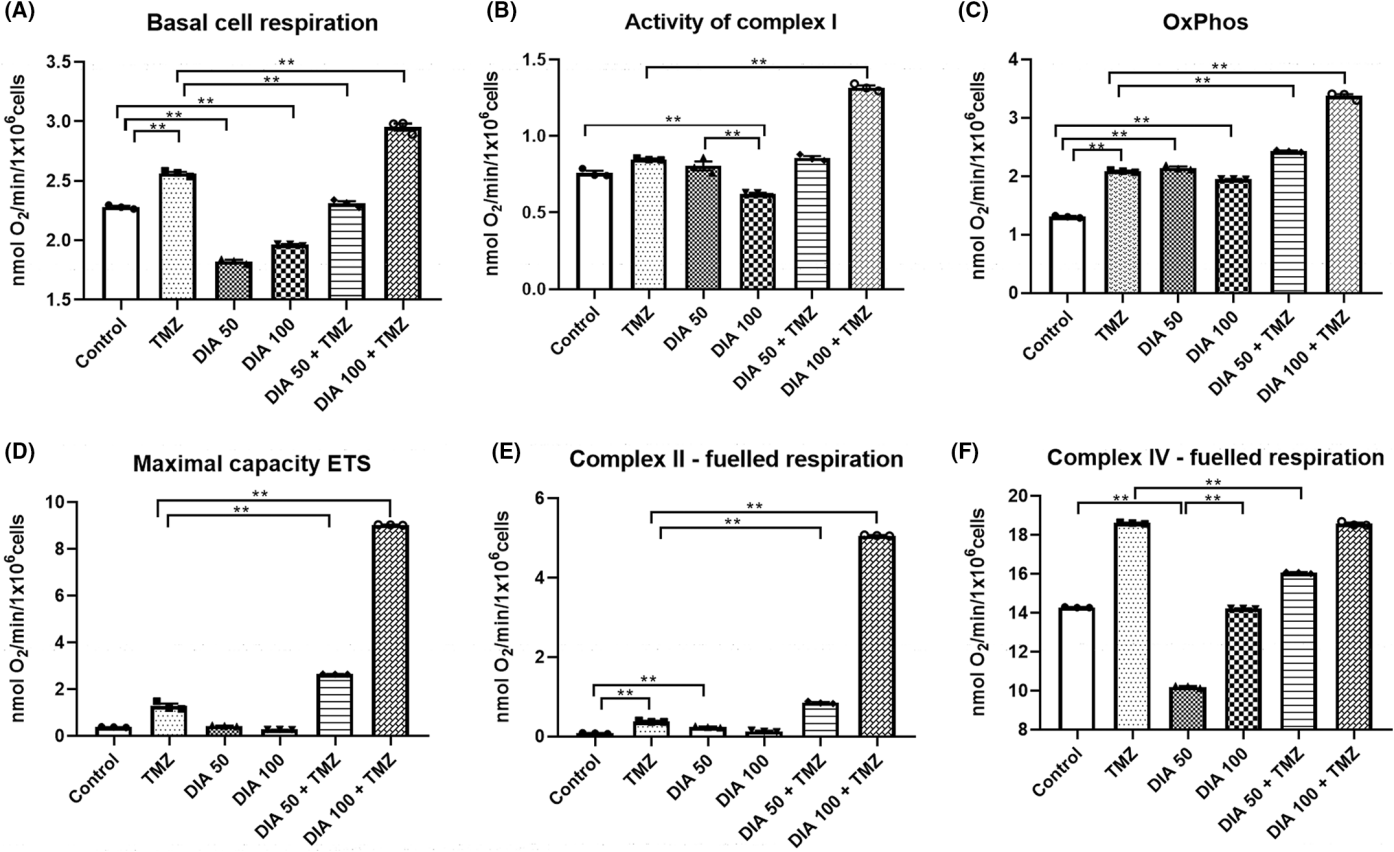
Measurements of oxygen consumption in U87 cells upon the treatment with TMZ and DIA alone, or in a combination. Basal cell respiration (A), the activity of complex I (B), the OxPhos rate (C), the maximal capacity of the electron transport system (ETS) (D), the rates of complex II‐fueled (E), and complex IV‐fueled respiration (F) were measured. Data are presented as mean ± S.E.M. *p < 0.01 and **p < 0.001.

The basal cell respiration was recorded in intact U87 cells (Figure 6A). DIA in both concentrations decreased, while TMZ increased the respiration rate in comparison with untreated U87 cells (*p* < 0.001). However, the combined treatment of TMZ and DIA showed bidirectional effects, decreasing the respiration in a lower concentration of DIA, and increasing the respiration rate in a higher concentration of DIA, in comparison with TMZ alone (*p* < 0.001). After adding digitonin to disrupt the cell membrane, and exogenous substrates fueling complex I respiration (Figure 6B), a significant decrease was observed in DIA 100 in comparison with DIA 50 and control (*p* = 0.001). However, DIA 100 in combination with TMZ showed a significant increase in the activity of complex I in comparison with TMZ alone (*p* < 0.001). The OxPhos rate is presented in Figure 6C. Treatment with TMZ or DIA in both concentrations led to an increase in respiration rate in comparison with control (*p* < 0.001). Moreover, TMZ in combination with DIA in both concentrations enhanced the respiration rate (*p* < 0.001), with a more pronounced effect in the group treated with a higher concentration of DIA. The uncoupler FCCP was used to determine the maximal capacity of the mitochondrial electron transport system (ETS) (Figure 6D). Results showed that DIA in combination with TMZ significantly increased the oxygen uptake in a concentration‐dependent manner (*p* < 0.001). The same trend was observed in the complex II‐fueled respiration rate, which was determined after the inhibition of complex I with rotenone (Figure 6E, *p* < 0.001). In addition, the rate of complex IV‐fueled respiration was evaluated (Figure 6F). Treatment with DIA 50 resulted in a significant decrease in oxygen consumption in comparison with DIA 100 and control (*p* < 0.001). While TMZ in combination with DIA 100 did not alter the oxygen consumption rate, in combination with DIA 50 a significant decrease was observed in comparison with TMZ alone (*p* < 0.001).

## 4 DISCUSSION

Symptom burden in GBM patients is an eminent challenge that requires the simultaneous use of several drugs. Polytherapy increases the complexity of therapeutic managing and thereby the risk of drug‐drug interactions.^31^ One such drug normally administered to patients who are under GBM treatment to combat symptoms of anxiety, status epilepticus, or chemotherapy‐induced emesis is diazepam.^9^, ^10^, ^11^ Interestingly, to the best of our knowledge, this is the first study reporting the combined effects of TMZ and DIA in GBM. Since there is no data on possible side effects of simultaneous administration, we believe it is critical to further evaluate the efficacy of TMZ in the presence of DIA.

In addition to the aforementioned effects, over the years DIA has gained an ongoing interest in the biological activity as an antitumor agent. As a first approach, the screening was carried out via MTT assay using U87 GBM cells to assess the viability inhibiting effects. Our results were in line with other studies, reporting the antiproliferative effects of DIA in various cancer cell lines, such as U87 GBM, C6 glioma, B16 melanoma, and neuroblastoma.^12^, ^20^, ^21^, ^32^ Moreover, DIA was shown to enhance the cytotoxicity of etoposide,^22^ anti‐CD95 antibody,^33^ hypericin,^23^ and lonidamine.^18^ Therefore, given the pronounced antiproliferative effects of DIA on its own, it prompted us to reckon that diazepam could enhance temozolomide‐induced cytotoxicity in GBM cells. However, despite the enhanced cytotoxicity in the combinatorial approach, the cooperative index indicated an antagonistic effect implying that DIA exposure of 72 h did not elicit any substantial enhancement of TMZ efficacy on simultaneous administration. Furthermore, inhibition of cell growth in long‐term colony formation assay corroborates the cooperative index, revealing that increased TMZ‐induced cytotoxicity by concomitant use of DIA is reversible, does not persist after drug withdrawal, and even leads to reduced TMZ efficacy in long‐term observation.

Few studies have focused on revealing the mechanism of antiproliferative effects of benzodiazepines in tumors. Diazepam is known to bind with relatively high affinity to both peripheral‐type (PBR, known as the translocator protein) and central‐type benzodiazepine receptors (CBR, known as a GABA‐A receptor).^34^, ^35^ Benzodiazepines appear to exert proapoptotic effects in tumor cells through PBR and regulation of mitochondrial transmembrane potential.^23^, ^35^ In addition, it is found that enhanced anion permeability in tumor cells, mediated via CBR, depolarizes their mitochondria and thereby elicits an apoptotic response.^36^, ^37^ Kallay et al.^36^ showed that benzodiazepine‐modulated CBR sensitized medulloblastoma cells to radiation and/or cisplatin, whereas Pomeranz Krummel et al.^37^ used melanoma cells in their study and noticed an enhanced susceptibility to radiation and/or an immune checkpoint inhibitor after modulation of CBR with benzodiazepines. In line with these results, we showed that U87 GBM cells underwent apoptosis following treatment with TMZ or DIA when administered alone, however, with no change in the extent of apoptosis upon the simultaneous administration. Therefore, it can be concluded that DIA does not enhance TMZ toxicity in simultaneous administration, but when administered individually impairs cell viability, inducing apoptosis in U87 GBM cells after 72 h.

Moreover, there is a lot of evidence that benzodiazepines, including DIA, interfere with cell cycle regulation and possess pro‐differentiating effects in a number of cell types.^38^ In micromolar concentrations, DIA elicited G0/G1 cell cycle arrest in human GBM independently of its specificity for different receptors,^32^ causing cells to remain in their quiescent stage. Having in mind that differentiating agents induce G1 arrest in tumor cells, it is possible that DIA possesses differentiating features.^21^ This explanation supplements morphological assessment regarding the DIA‐induced phenotypic alterations in U87 cells compatible with a more differentiated phenotype, displaying a star shape with neurite‐like protrusions, along with the rearrangement of intermediate filament vimentin.

The capability of cell motility and invasion is important in tumor malignancy and recurrence.^39^, ^40^ The morphological changes upon the treatments could be the reason for an increased TCCF of vimentin in all experimental groups, increasing the signal intensity on a smaller cell area.^41^ Bearing in mind that the cells upon the treatment with DIA displayed shrinkage with neurite‐like protrusions, thus had larger perimeters, this could be an explanation for high motility in wound healing assay upon the treatment with DIA 50, since large total perimeter half ratio values typically indicated fast‐moving cells.^42^

Temozolomide, as the standard adjuvant chemotherapy in patients with GBM, exerts its effects through the ability to methylate DNA at the N^7^ or O^6^ positions of guanine residues. This causes mispairing with thymine during replication, alerting DNA mismatch repair, and finally resulting in G2/M cell cycle arrest, occurring in the second cell cycle following treatment, and ultimately apoptosis.^43^ Given that DIA triggers G0/G1 cell cycle arrest in human GBM cells,^32^ which precedes the S phase, thus blocking the DNA replication, it attenuates the TMZ efficacy and should not be administered together. Hence, we did not observe differences between TMZ alone and in combination with DIA.

PBRs are located at the mitochondrial outer membrane, which has naturally led to an interest in cellular respiration.^35^ It has been reported that peripheral benzodiazepines modulate mitochondrial function, through inhibition of mitochondrial respiratory control, thus decreasing the oxygen consumption in mouse neuroblastoma cells.^44^ In addition, it has been shown that this respiration‐inhibiting effect of PBR ligands occurs only at high concentrations.^38^ Therefore, by using DIA in micromolar concentrations, our aim was to interfere with the mitochondrial respiratory complexes in the hope of causing perturbation in energy demand. Our data are consistent with previous research showing an inhibitory effect of DIA on basal cell respiration and the activities of complexes I and IV. On the contrary, TMZ treatment was shown to stimulate mitochondrial respiration. Since the measurement of oxygen consumption was normalized to the number of viable cells, this activity could be attributed to its ability to increase the number of mitochondria per cell.^45^, ^46^ Nevertheless, the combination treatment in U87 GBM cells seems to make mitochondria more active. Bearing in mind that U87 cells rely primarily on glycolysis rather than OxPhos,^47^ we could speculate that TMZ in the presence of DIA induced a shift in metabolic phenotype. Desai et al.^48^ also noticed a clear shift of U87 cells toward OxPhos upon the combined treatment of TMZ and biochanin A.

Given that high glycolytic activity with low mitochondrial function contributes to GBM tumorigenicity and aggressiveness,^47^ restoring the mitochondrial function in GBM would decrease metabolic intermediates and hinder proliferation.^48^ In addition, mitochondrial transplantation into U87 xenograft tumors reactivated the mitochondrial apoptotic pathway, inhibited tumor growth, and augmented GBM radiosensitivity.^49^ Hence, these results suggest that concomitant use of TMZ and DIA attenuated the Warburg effect in U87 GBM cells. Moreover, an increased OxPhos level substantially contributes to the generation of reactive oxygen species, thus activating mitochondrial apoptotic machinery and, consequently, causing a detrimental cytotoxic response.^50^

Altogether, in contrast to the broad data available on the beneficial effects of these drugs on their own as antitumor agents, little is known about their combined effect on the tumorigenicity of GBM. Comparing our results with data reported that DIA elicits cell cycle arrest in the G0/G1 phase and favors senescence reveals that DIA diminishes TMZ efficacy in concomitant use in the treatment of GBM. However, due to its great potency to hinder GBM proliferation and metabolism, it could be considered using DIA as maintenance therapy after TMZ cycles.

## CONFLICTS OF INTEREST

The authors declare they have no competing interests.

## Supporting information


Appendix S1

## Data Availability

The data that support the findings of this study are available from the corresponding author upon reasonable request.
