# Supplementary material for: Diazepam diminishes temozolomide efficacy in the treatment of U87 glioblastoma cell line
Source: CNS Neurosci Ther. 2022 Jun 15;28(9):1447–57. doi: 10.1111/cns.13889 (PMC9344091; doi:10.1111/cns.13889)
Supplement: Supplementary file 1 — Appendix S1 [file CNS-28-1447-s001.docx]

**DIAZEPAM DIMINISHES TEMOZOLOMIDE EFFICACY IN THE TREATMENT OF U87 GLIOBLASTOMA CELL LINE**

Drljača Jovana, Popović Aleksandra, Bulajić Dragica, Stilinović Nebojša, Vidičević Novaković Sašenka, Sekulić Slobodan, Milenković Ivan, Ninković Srđan, Ljubković Marko, Čapo Ivan; Correspondence: [jovana.drljaca@uns.ac.rs](mailto:jovana.drljaca@uns.ac.rs)

**SUPPORTING INFORMATION**

**Immunofluorescence**

Cells were harvested and plated in a 24-well plate (5x10^4^ cells/well) containing glass coverslips. Following the treatment, cells were washed out with phosphate buffer saline, fixed with 4% paraformaldehyde, and permeabilized with 0.3% Triton X-100. After the incubation with blocking solution 10% normal goat serum, the following primary antibodies were added: anti-vimentin (rabbit monoclonal, 1:200 dilution, ab92547, Abcam), and anti-Bcl-2 (rabbit monoclonal, 1:500 dilution, ab108346, Abcam). Afterwards, the slides were incubated with secondary anti-rabbit antibodies linked to Alexa Fluor^®^ 488 (for vimentin, ab150077, Abcam) or Alexa Fluor^®^ 555 (for Bcl-2, ab150078, Abcam) (goat, polyclonal, both), and the nuclei were counterstained with DAPI (ab104139, Abcam).

Images were analyzed using a fluorescence microscope (Leica DMLB 100T) and five random fields were photographed on Leica MC 190 camera at 200x magnification. All morphometric analyses were performed using *Fiji* image software. Quantification of vimentin expression was expressed as corrected total cell fluorescence (CTCF). Upon outlining around 10 cells per image, the total area of selected cells and the mean fluorescence (integrated density) were measured, along with several adjacent background readings. The corrected total cell fluorescence (CTCF) was calculated as integrated density – (area of selected cell × mean fluorescence of background readings). The percentage of anti-Bcl-2 positive tumor cells (%(Bcl-2)+ cells) was calculated in five microscopic fields at x200 magnification.

**Quantitative reverse transcription and PCR (RT-PCR)**

To determine the expression of certain genes in the continuous human glioblastoma U87 cell line, cells were seeded in a T75 flask and, after reaching a confluence of 80%, treated with TMZ or/with DIA for 72 h. Afterwards, the cells were trypsinized, collected and centrifuged at 300 RCF for 10 min, then the cell pellet was washed in PBS and centrifuged again in the same manner. To isolate RNA, cell samples were lysed with trizole (TRIzol Reagent, Invitrogen Ambion), proteins were precipitated with chloroform, while isopropyl alcohol was added to the supernatant to precipitate RNA. The precipitate was resuspended in 20 μl of nuclease-free water. In addition, DNAse treatment was performed using the Turbo DNA free^TM^ kit (Ambion®, Thermo Fisher Scientific) according to the manufacturer's instructions. The concentration of isolated RNA was measured using a Qubit 3 fluorometer (Invitrogen) with an adequate RNA kit (Qubit^TM^ RNA Broad Range Assay, Thermo Fisher Scientific). The uniform amount of RNA (2 μg) from all tested samples was converted to cDNA by using the High-Capacity cDNA Reverse Transcription Kit (Applied Biosystems) according to the manufacturer’s instruction.

The expression of the genes *BAX*, *Bcl-2*, and *TBP* was quantified on Real-Time PCR 7500 Fast (Applied Biosystems) detection system using the Power SYBR™ Green PCR Master Mix (Applied Biosystems) in the presence of an aliquot of 2.5 µl of the cDNA and specific primers at the final concentration of 500 nM (at reaction volume of 12.5 µl). The thermal profile used for amplification was 50 °C for 2 min and 95 °C for 10  min, followed by 40 cycles of 95 °C for 15 s, 60 °C for 1 min. At the end of the amplification phase a melting curve analysis was carried out according to the dissociation stage data and reactions with a single peak at expected temperature melting (Tm) were considered for further analysis.

The relative quantification of mRNA was performed as a value relative to an endogenous control *TBP*, further in comparison to the control group using the 2^-ΔΔCt^ method. All primers were designed by Primer Express 3.0 (Applied Biosystems). The characteristics of the primers used in this study have been summarized in **Table S1**. Relative quantification of each gene was conducted in triplicate. Negative non-template controls were included in each set of reactions.

***Table S1.*** The primers sequences used for real-time PCR analysis of apoptosis level in U87 glioblastoma cells.

| **Gene** | **Accession code** | **Primers** | **Primer length** | **Prod. length** |
| --- | --- | --- | --- | --- |
| ***Bcl2*** | [NM_000633.3](https://www.ncbi.nlm.nih.gov/entrez/viewer.fcgi?db=nucleotide&id=1830949192) | F: 5'- TCCCTCGCTGCACAAATACTC -3'  R: 5'- ACGACCCGATGGCCATAGA -3' | 21 bp  19 bp | 72 bp |
| ***BAX*** | [NM_004324.4](https://www.ncbi.nlm.nih.gov/entrez/viewer.fcgi?db=nucleotide&id=1674986022) | F: 5'-CTGCAGAGGATGATTGCCG -3'  R: 5'- TGCCACTCGGAAAAAGACCT -3' | 19 bp  20 bp | 63 bp |
| ***TBP*** | [NM_003194.5](https://www.ncbi.nlm.nih.gov/entrez/viewer.fcgi?db=nucleotide&id=1519313030) | F: 5'- GAGCTGTGATGTGAAGTTTCC -3'  R: 5'- TCTGGGTTTGATCATTCTGTAG -3' | 21 bp  22 bp | 118 bp |
